# Associations of Hypothyroxinemia With Risk of Preeclampsia–Eclampsia and Gestational Hypertension

**DOI:** 10.3389/fendo.2021.777152

**Published:** 2021-11-04

**Authors:** Xiujuan Su, Yang Liu, Guohua Li, Xiaosong Liu, Shijia Huang, Tao Duan, Qiaoling Du

**Affiliations:** ^1^ Clinical Research Centre, Shanghai Key Laboratory of Maternal Foetal Medicine, Shanghai First Maternity and Infant Hospital, School of Medicine, Tongji University, Shanghai, China; ^2^ Department of Obstetrics, Shanghai First Maternity and Infant Hospital, School of Medicine, Tongji University, Shanghai, China; ^3^ Department of Reproductive Immunology, Shanghai First Maternity and Infant Hospital, School of Medicine, Tongji University, Shanghai, China

**Keywords:** hypothyroxinemia, cohort study, hypertension disorders during pregnancy, preeclampsia–eclampsia, gestational hypertension (GH)

## Abstract

**Objective:**

To investigate the association between hypothyroxinemia and the risk of preeclampsia–eclampsia and gestational hypertension.

**Design:**

Historical cohort study.

**Methods:**

The study included pregnant individuals who delivered live-born singletons and had at least one thyroid function assessment during pregnancy at a tertiary hospital. Hypothyroxinemia was defined as thyroid-stimulating hormone (TSH) levels within the normal reference range and free thyroxine (FT4) levels lower than the tenth percentile. Risk ratios (RRs) with 95% confidence intervals (95% CIs) for preeclampsia–eclampsia and gestational hypertension between women with and without a diagnosis of hypothyroxinemia during pregnancy were estimated using a generalized estimating equation model.

**Results:**

A total of 59,463 women with live-born singletons were included in the analysis. Logistic regression models with restricted cubic spline suggested that there was a U-shaped association between FT4 levels and preeclampsia–eclampsia risk. Compared with euthyroid women, those with hypothyroxinemia had an increased risk of preeclampsia–eclampsia (RR = 1.16, 95% CI: 1.02–1.31), and the risk increased with the increasing severity of hypothyroxinemia (*p* for trend < 0.001). Moreover, persistent hypothyroxinemia from the first to second trimesters was associated with an increased risk of preeclampsia–eclampsia (RR = 1.37, 95% CI: 1.03–1.83), especially for women with severe hypothyroxinemia (RR = 1.70, 95% CI: 1.12–2.58). In contrast, there was no association between hypothyroxinemia and gestational hypertension.

**Conclusion:**

Our study suggested that hypothyroxinemia was only associated with an increased risk of preeclampsia–eclampsia, especially in women with persistent hypothyroxinemia in the first half of pregnancy. Analyses of the associated risk of gestational hypertension with hypothyroxinemia were not significant.

## Introduction

Hypothyroxinemia is defined as a decrease in serum free thyroxine (FT4) levels with thyroid-stimulating hormone (TSH) levels within normative reference limits, and affects up to 10% of women of reproductive age (1, 2). In the context of pregnancy, human chorionic gonadotropin (hCG) acting as TSH-like agonist on thyroid gland (3), the prevalence of thyroid dysfunction is more common among pregnant women (2, 4). Previous studies have consistently elucidated that maternal hypothyroxinemia is associated with a delayed neuropsychological development of the offspring (5–8), while lines of evidence for the relationship between hypothyroxinemia and pregnancy outcomes have been limited and conflicting.

It has been reported that thyroid hormones play a role in trophoblast proliferation and differentiation and trophoblast invasion of the decidual and decidual angiogenesis (9), which is a main underlying pathophysiology for hypertension disorders during pregnancy (HDP) (10). HDP is an important cause of death and complications for the mother and baby, especially its subtypes of preeclampsia–eclampsia. In some (11–14) but not all studies (15–17), women with lowered thyroxine levels had a greater risk of HDP. Most previous studies were limited by insufficient sample size and power, and were not able to differentiate the association by subtypes of HDP, including preeclampsia–eclampsia and gestational hypertension, or by gestational age (12, 18). In addition, there is also a positive relationship between serum TSH levels and both systolic and diastolic blood pressure (19, 20). Previous studies on hypothyroxinemia and preeclampsia–eclampsia generally did not take TSH levels into account. Furthermore, thyroid peroxidase antibody (TPOAb) had an effect on the trajectory of thyroid hormone during pregnancy (21) and might be associated with the risk of pregnancy complications (22). In this context, we performed a historical cohort study to explore the association of hypothyroxinemia with preeclampsia–eclampsia and gestational hypertension, considering TSH and TPOAb status. We hypothesized that women with hypothyroxinemia would have a greater risk of preeclampsia–eclampsia or gestational hypertension than euthyroid women, and trimester-specific effects might exist (23, 24).

## Methods

### Study Population and Data Source

We conducted a historical cohort study at the Shanghai First Maternity and Infant Hospital in China. All pregnancies registered in the electronic medical registers between January 2014 and September 2019 were included in this study.

All women were invited to provide personal sociodemographic and health information at the initial antenatal appointment, including age at conception, residence of origin, parity, height (in centimeters), and weight before pregnancy (in kilograms). The prepregnancy body mass index (BMI) was calculated by dividing the weight (in kilograms) by the height (in meters squared). According to recommendations of appropriate BMI for Asian population by the World Health Organization (WHO), prepregnancy BMI was categorized as underweight (<18.5 kg/m^2^), optimal weight (18.5–23 kg/m^2^), overweight (23–27.5 kg/m^2^), and obesity (≥27.5 kg/m^2^). Gestational age was assessed based on the date of the last menstrual period and the results of early ultrasound. We also asked the women if they had a history of chronic diseases before pregnancy, including hypertension, diabetes, and thyroid dysfunction before pregnancy.

**Figure 1:**
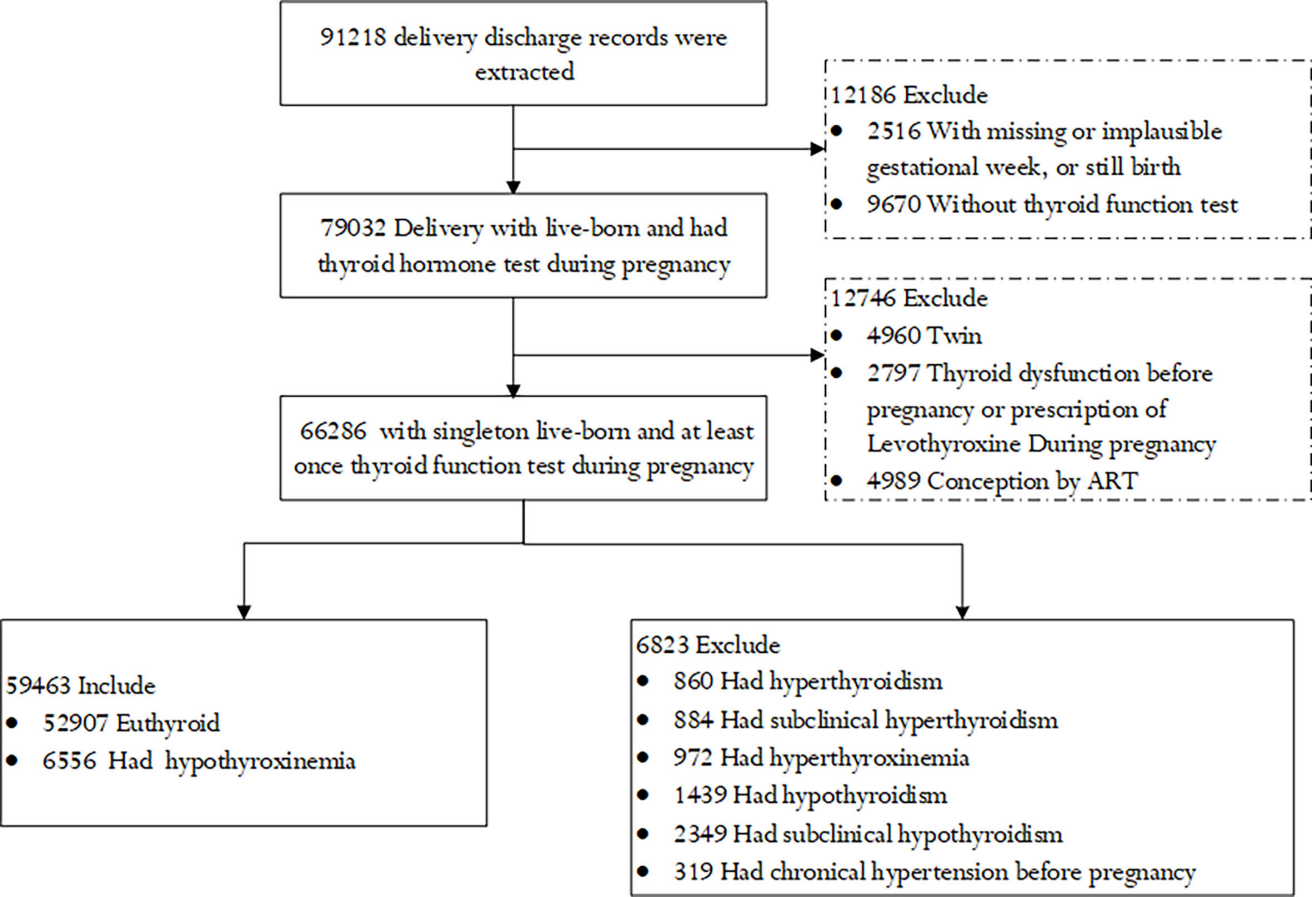
Flowchart of the study population.

We excluded women who had pregnancies that ended with stillbirth, who had implausible gestational weeks, or did not have a thyroid function assessment during pregnancy. Women who conceived by assistant reproductive techniques (ART), were pregnant with twins, had a diagnosis history of thyroid dysfunction, had chronic hypertension or diabetes, or had new-onset maternal thyroid dysfunction during pregnancy except hypothyroxinemia were also excluded from the study. Finally, a total of 59,463 women with live-born singletons were included in the study (**Figure 1**).

### Measurement of Thyroid Function

Fasting blood samples were collected at antenatal visits and were centrifuged (10 min with rethawing cycles at 3,000 rpm) to obtain serum levels. Serum TSH, FT4, and TPOAb were measured [ADVIA Centaur instruments and kits (Siemens, Munich, Germany)]. According to the guidelines published by the American Thyroid Association in 2017 (25), we built the laboratory- and trimester-specific reference ranges for TSH and FT4 levels (**Table S1**). The criteria used to diagnose hypothyroxinemia were a TSH level in the normal reference range and a FT4 level lower than the 10th percentile. Women diagnosed with hypothyroxinemia of any two trimesters were defined as patients with persistent hypothyroxinemia. We also categorized the severity of hypothyroxinemia as mild (from the 5th to 10th percentile), moderate (from the 2.5th to 5th percentile), and severe (lower than the 2.5th percentile). When the TSH and FT4 levels were both in the normal reference ranges, women were defined as euthyroid. For women with more than one thyroid function test, we kept the earliest thyroid function test value by trimester in the final analysis. A TPOAb level greater than 60 U/ml was regarded as positive according to the manufacturer-defined cutoff.

### Diagnosis of Preeclampsia–Eclampsia and Gestational Hypertension

The diagnosis of preeclampsia–eclampsia and gestational hypertension was identified from electronic discharge records. The diagnosis criteria followed the guidelines of the Chinese Society of Obstetrics and Gynecology during the study period (26). Gestational hypertension was defined as the onset of hypertension after 20 weeks of gestation with a systolic blood pressure of at least 140 mmHg and/or a diastolic blood pressure of at least 90 mmHg. Preeclampsia was defined as the elevated blood pressure and proteinuria, or (in the absence of proteinuria) new-onset hypertension with any of the following conditions: (1) impaired organ; (2) abnormalities of the blood, digestive, or nervous systems; or (3) placenta–fetal involvement. Preeclampsia could be subclassified into early-onset preeclampsia (with delivery at <34+0 weeks of gestation) and late-onset preeclampsia (with delivery at ≥34+0 weeks of gestation). Eclampsia was diagnosed based on a preeclampsia diagnosis combined with seizures without explained reasons.

### Statistical Analysis

Continuous variables are presented as the means with standard deviations or medians with interquartile ranges (IQRs) according to their distribution. Categorical variables are presented as counts with percentages. The baseline characteristic differences between euthyroid women and women with hypothyroxinemia were estimated using *t*-tests, or nonparametric tests, or chi-squares tests, or Fisher’s exact tests, as appropriate.

Logistic regression model with restricted cubic spline (RCS) was used to estimate the nonlinear relationship between FT4 levels and the risk of preeclampsia–eclampsia and gestational hypertension. Due to its skewed distribution, the Ln-transformed value of the FT4 levels was used in the model. We set the 50th percentile as a reference and four knots (the 25th, 50th, 75th, and 90th percentiles) in the model. Risk ratios (RRs) and 95% confidence intervals (95% CIs) were calculated using generalized estimating equation (GEE) model, with adjustment for the following potential confounders: advanced age (≥35 years) at pregnancy (yes or no), prepregnancy BMI category (underweight, <18.5 kg/m^2^; optimal weight 18.5–23 kg/m^2^, overweight 23–27.5 kg/m^2^, or obesity ≥27.5 kg/m^2^), parity (parous or nulliparous), and fetal sex (boy or girl). The residence of origin (Shanghai, other areas) was also included in the multivariable analyses as an index of nutrition and physical activity habits. We do not have information on smoking status, which might be a confounder in the present study, while we believed that smoking status would have little influence on the association of FT4 and pregnancy-induced hypertension in the study population as a previous study indicated a low prevalence of both active and passive smoking (1.2% and 7.8%) among pregnant women in Shanghai (27). Ln-transformed TSH levels were also included in the model as continuous variables. In addition, our previous study indicated that serum ferritin might also modify the association of FT4 levels and pregnancy complications (28). Serum ferritin was also included in the model as a continuous variable. Serum ferritin was measured by a UniCel DxI 800 immunology analyzer and kits (Beckman Coulter, California, USA) at the institute. We also performed tests for linear trends by entering the severity of hypothyroxinemia as a continuous variable in the model.

We performed several sensitivity analyses to evaluate the robustness of the results from our primary analyses. First, we conducted an analysis by stratifying on the diagnosis time of hypothyroxinemia (only the first trimester, only the second trimester, from the first to second trimester). Second, we restricted the analysis to women with optimal prepregnancy BMI, negative TPOAb, and TSH levels ranging from the 2.5th percentile to 2.5 mU/L, as these factors might bias or modify the associations considered. Third, a propensity score matching (PSM) method was used to balance the differences in baseline characteristics (29). The propensity score for hypothyroxinemia was estimated using a logistic regression model with all covariates in the main analysis as independent variables. The PSM was performed with 1:1 matching by nearest-neighbor matching, with a caliper width equal to 0.2 and no knots were used for those continuous variables. No adjustment RRs (95% CIs) were estimated after PSM using the logistic regression model.

We used “mkspline”, “xblc”, and “logit” routines of Stata 16 MP for the RCS. We performed propensity score matching using SPSS version 25.0 software (SPSS Inc., Chicago, IL, USA). All other statistical analyses were performed using SAS software (version 9.4). The precision of risk estimates is described using 95% confidence intervals. Interpretation of the results was mainly based on the strength of the adjusted RR (regardless of whether the 95% CIs included the null).

## Results

### Basic Characteristics of the Study Population

A total of 59,463 women with 79,456 observations were included in the study, and of those, 53,336 (89.7%) had only one thyroid function test. There were 6,556 women who received a diagnosis of hypothyroxinemia, with 236 (3.6%) and 321 (5.08%) women having preeclampsia–eclampsia and gestational hypertension, respectively. **Table 1** shows the characteristics of the study population according to with or without a hypothyroxinemia diagnosis. Women with hypothyroxinemia were more likely than euthyroid women to be parous (31.47% *vs.* 24.82%), have an advanced age (17.37% *vs.* 12.85%), be TPOAb positive (11.96% *vs.* 8.93%), and have prepregnancy overweight or obesity (21.04% *vs.* 27.11%), but women with hypothyroxinemia were less likely to be of Shanghai origin (27.53% *vs.* 31.55%). **Table 2** presents the medians with IQRs of TSH and FT4 concentrations for all observations. The median FT4 were 12.77 (12.12–13.55) pmol/L and 16.11 (14.85–17.56) pmol/L, and the median TSH were 1.55 (1.04–2.16) mIU/L and 1.30 (0.79–1.91) mIU/L for hypothyroxinemia and euthyroid women, respectively.

**Table 1:** The characteristics of the study population.

Characteristics	Euthyroid (*n* = 52,907)	Hypothyroxinemia (*n* = 6,556)	*p-*value
Advanced age (≥35 years old)			<0.001
Yes	6,798 (12.85)	1,138 (17.37)	
No	46,109 (87.15)	5,418 (82.64)	
Prepregnancy BMI			<0.001
Underweight (<18.5 kg/m^2^)	7,569 (14.31)	701 (10.69)	
Optimal weight (18.5–23 kg/m^2^)	34,205 (64.65)	4,078 (62.20)	
Overweight (23–27.5 kg/m^2^)	8,918 (16.86)	1,432 (21.84)	
Obesity (≥27.5 kg/m^2^)	2,215 (4.19)	345 (5.26)	
Ethnicity			0.114
Minority	1,435 (2.71)	200 (3.05)	
Majority	51,472 (97.29)	6,356 (96.95)	
Shanghai origin			<0.001
Yes	36,213 (68.45)	4,751 (72.47)	
No	16,694 (31.55)	1,805 (27.53)	
Parity			<0.001
Nulliparous	39,774 (75.18)	4,493 (68.53)	
Parous	13,133 (24.82)	2,063 (31.47)	
Positivity TPOAb			<0.001
Yes	4,727 (8.93)	784 (11.96)	
No	48,180 (91.07)	5,772 (88.04)	
Gestational age at thyroid measurement (days)*	85 (60–114)	103 (64–144)	<0.001

BMI, body mass index; TPOAb, thyroid peroxidase antibody. *Median (interquartile ranges).

**Table 2:** Description of FT4 and TSH levels (medians, interquartile range).

	No. of observations	FT4 (pmol/L)	TSH (mIU/L)
Hypothyroxinemia	9,491	12.77 (12.12–13.55)	1.55 (1.04–2.16)
Mild	4,641	13.02 (12.72–13.93)	1.52 (1.03–2.12)
Moderate	2,316	12.42 (12.17–13.37)	1.53 (1.02–2.16)
Severe	2,534	11.82 (11.35–12.57)	1.63 (1.10–2.25)
Euthyroid	69,965	16.11 (14.85–17.56)	1.30 (0.79–1.91)

### Association of FT4 Levels and the Risk of Preeclampsia–Eclampsia and Gestational Hypertension

**Figure 2:**
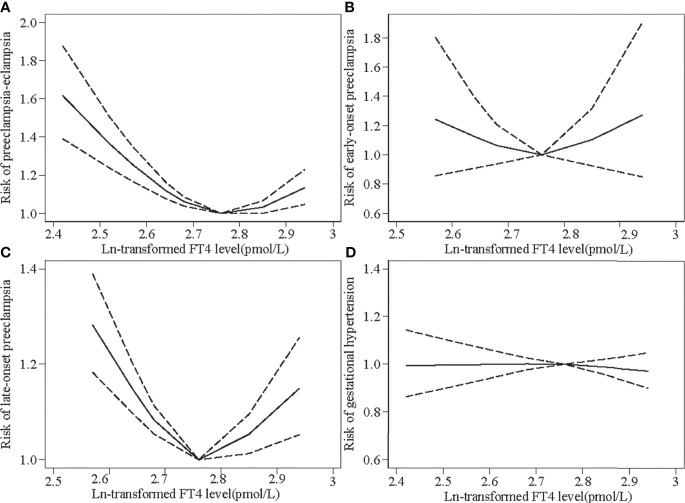
Restricted cubic spline models for Ln-transformed free thyroxine (FT4) levels and the risks of preeclampsia–eclampsia (PEE) and gestational hypertension (GH). Knots were placed at the 25th, 50th, 75th, and 90th percentiles. The black line indicates risk ratios for PEE and GH; dashed gray lines are the 95% confidence intervals. **(A)** Ln-transformed FT4 levels and the risk of overall PEE. **(B)** Ln-transformed FT4 levels and the risk of early-onset preeclampsia. **(C)** Ln-transformed FT4 levels and the risk of late-onset preeclampsia. **(D)** Ln-transformed FT4 levels and the risk of GH.

Overall, FT4 levels showed a U-shaped relationship with preeclampsia–eclampsia, with a higher risk at lower and higher FT4 levels during pregnancy compared with a median concentration of approximately 15.80 pmol/L (**Figure 2A**). The ORs (95% CIs) of preeclampsia–eclampsia across the 1st (11.25 pmol/L), 5th (12.42 pmol/L), 10th (13.06 pmol/L), and 90th (18.92 pmol/L) percentiles were 1.62 (95% CI: 1.39–1.88), 1.37(95% CI: 1.24–1.51), 1.26 (95% CI: 1.17–1.35), and 1.13 (95% CI: 1.05–1.23), respectively. We observed similar U-shaped associations for early-onset preeclampsia and late-onset preeclampsia (**Figures 2B, C**). FT4 levels did not seem to be associated with the risk of gestational hypertension (**Figure 2D**).

### Association of Hypothyroxinemia and the Risk of Preeclampsia–Eclampsia and Gestational Hypertension

**Figure 3:**
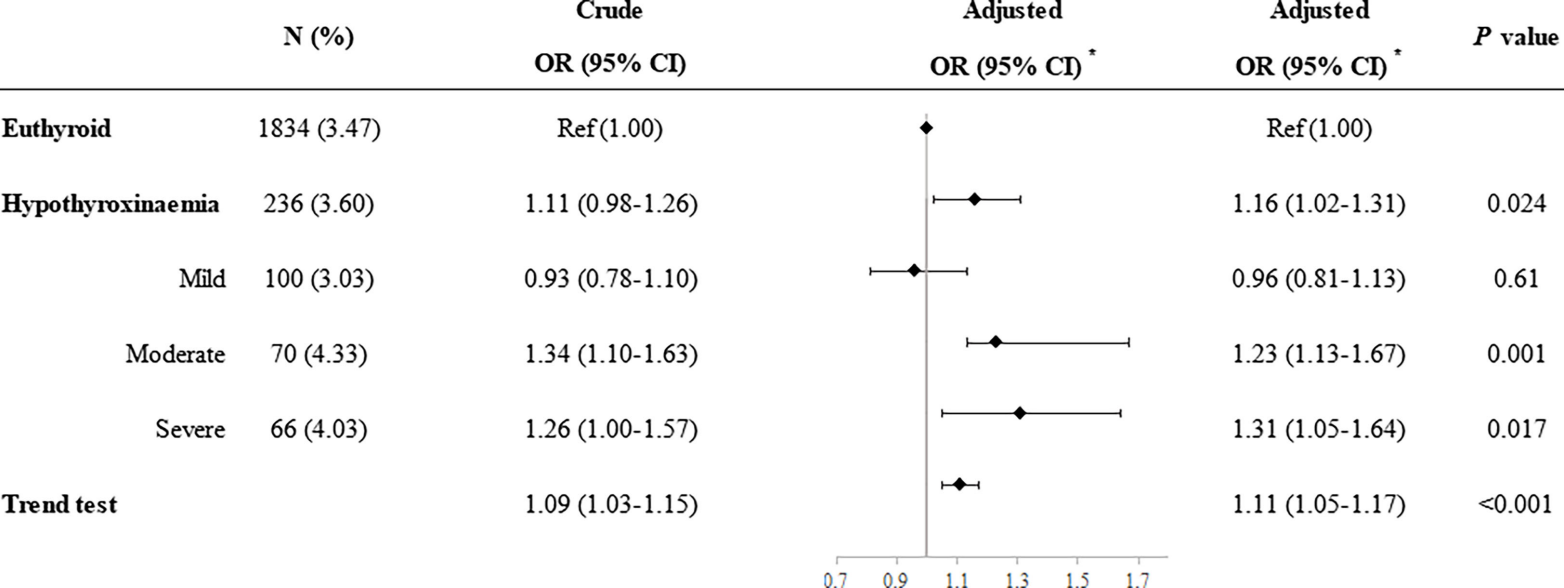
Association of hypothyroxinemia and overall risk of preeclampsia–eclampsia. *Adjusted for parity, age, prepregnancy BMI, fetus sex, residence of origin, serum ferritin, thyrotropin level, and TPOAb status.

**Figure 4:**
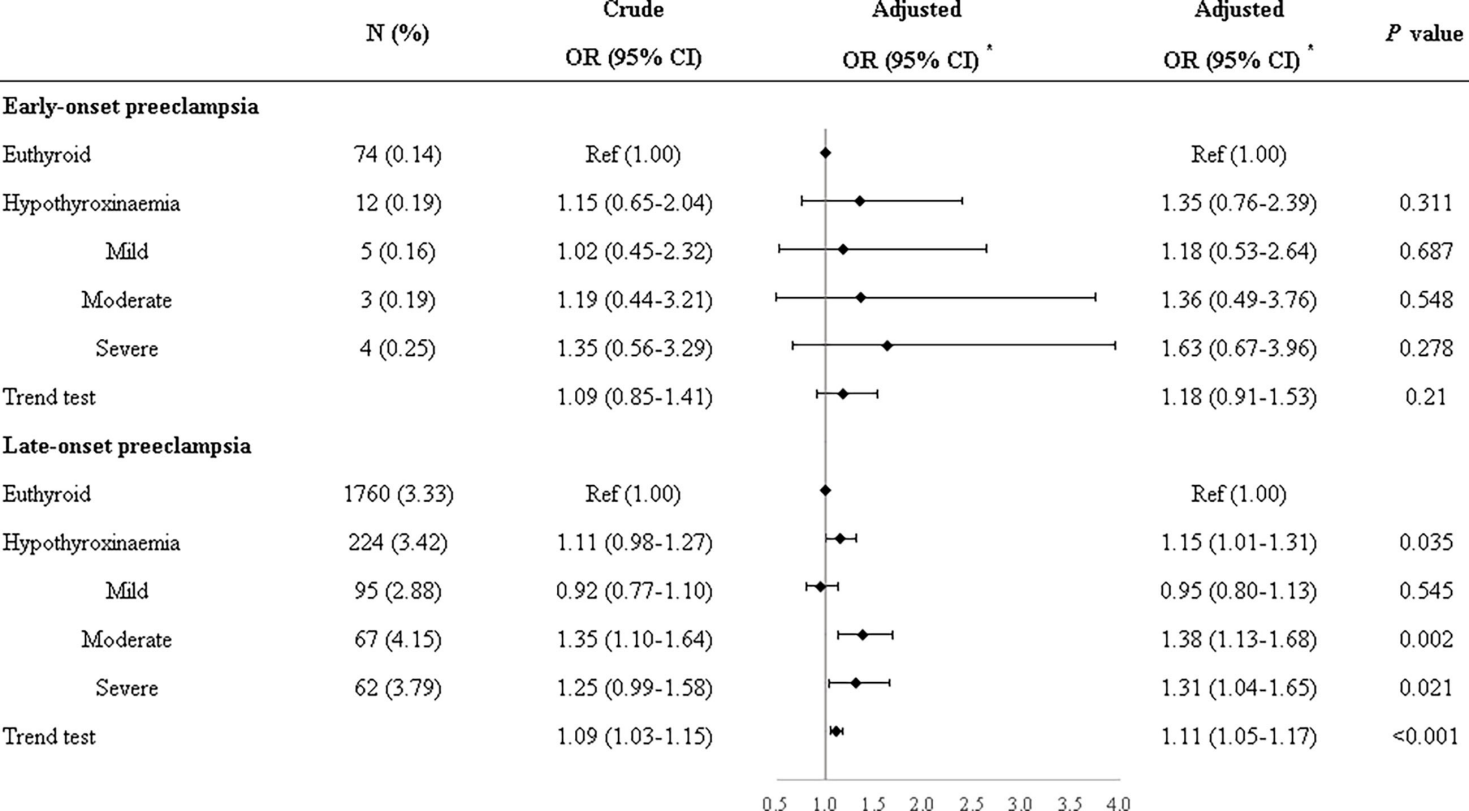
Associations of hypothyroxinemia and risk of early-onset preeclampsia–eclampsia and late-onset preeclampsia–eclampsia. *Adjusted for parity, age, prepregnancy BMI, fetus sex, residence of origin, serum ferritin, thyrotropin level, and TPOAb status.

**Figure 5:**
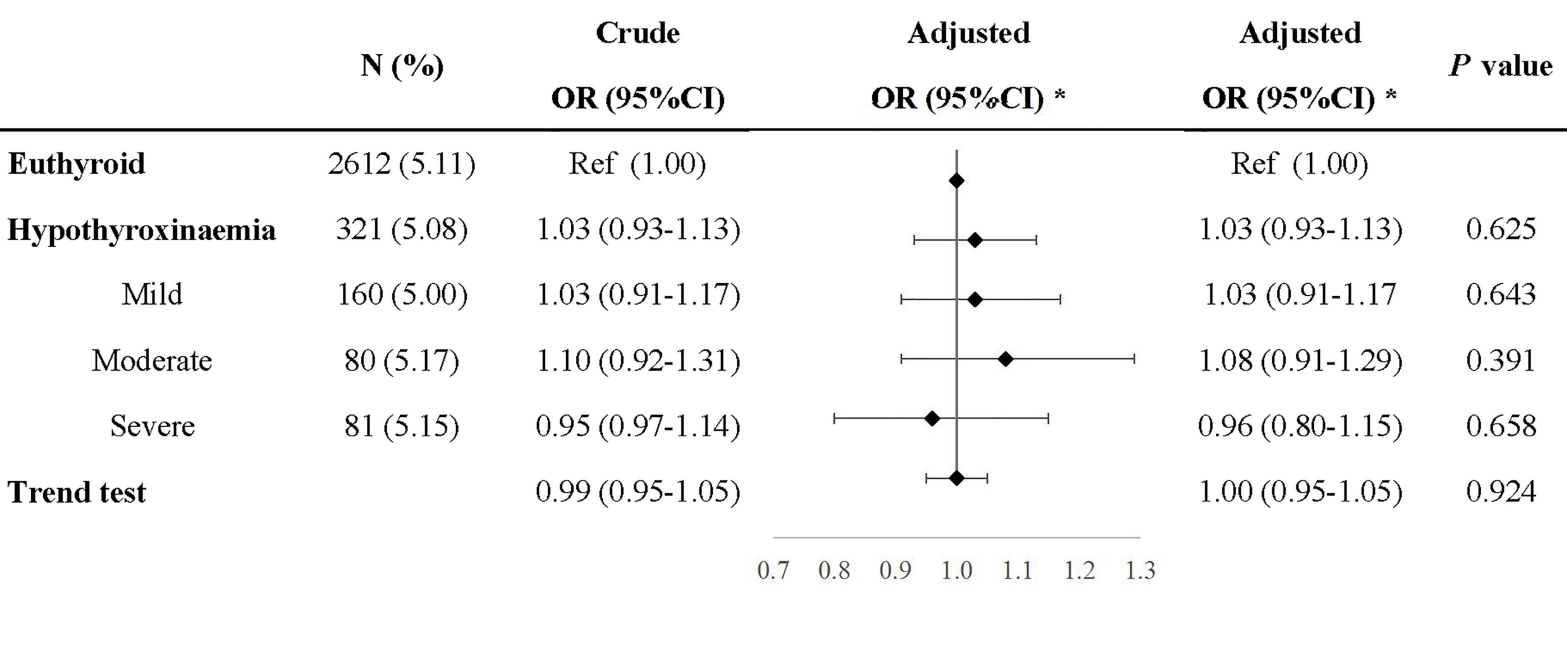
Association of hypothyroxinemia and risk of gestational hypertension. *Adjusted for parity, age, prepregnancy BMI, fetus sex, residence of origin, serum ferritin, thyrotropin level, and TPOAb status.

Compared with euthyroid women, women with hypothyroxinemia had an increased risk of preeclampsia–eclampsia, with a RR of 1.16 (95% CI: 1.02–1.31, *p* = 0.024) (**Figure 3**). The adjusted RR for preeclampsia–eclampsia was 0.96 (95% CI: 0.81–1.13, *p* = 0.610) for women with mild hypothyroxinemia, 1.23 (95% CI: 1.13–1.67 *p* = 0.001) for women with moderate hypothyroxinemia, and 1.31 (95% CI: 1.05–1.64, *p* = 0.017) for women with severe hypothyroxinemia. The linear trend tests indicated a dose–response relationship between hypothyroxinemia and preeclampsia–eclampsia risk (*p* for trend <0.001) (**Figure 3**). Both estimates were consistent with risk ratios mentioned above when evaluating the association of hypothyroxinemia with early-onset preeclampsia and late-onset preeclampsia, separately, although the width of the confidence interval for early-onset preeclampsia risk became broader and included the null due to the limited sample size (**Figure 4**). The estimates did not suggest an increased risk of gestational hypertension overall (RR = 1.03, 95% CI: 0.93–1.13, *p* = 0.625) or by severity of hypothyroxinemia (**Figure 5**).

### Sensitivity Analyses

We performed sensitivity analyses by stratifying on the time of hypothyroxinemia diagnosis to examine the potential time window of hypothyroxinemia for the risk of preeclampsia–eclampsia and gestational hypertension. Compared with euthyroid women, the adjusted RR of preeclampsia–eclampsia was 1.37 (95% CI: 1.03–1.83, *p* = 0.031) for women who presented hypothyroxinemia in both the first and second trimesters, 1.08 (95% CI: 0. 91–1.27, *p* = 0.389) for women with hypothyroxinemia only in the first trimester, and 1.12 (95% CI: 0.96–1.30, *p* = 0.165) for women with hypothyroxinemia only in the second trimester (**Table S2**). Women diagnosed with severe hypothyroxinemia had a greater risk of preeclampsia–eclampsia than euthyroid women, and the adjusted RR was up to 1.70 (95% CI: 1.12–2.58, *p* = 0.013). Generally, consistent results were observed when refining the analyses among women with optimal prepregnancy BMI, who were TPOAb negative, or had TSH levels lower than 2.5 mIU/L (**Tables S3**–**S5**). Sensitivity analyses based on PSM also yield estimates generally consistent with those from the main analysis, accounting for the broader of confidence interval (**Table S6**).

We reran the same sensitivity analyses for the association of hypothyroxinemia and gestational hypertension risk. There was no association between hypothyroxinemia and gestational hypertension in any of these analyses (data available at request).

## Discussion

Our study showed that there was a U-shaped association between FT4 levels and preeclampsia–eclampsia risk. This also suggested that the FT4 level within the normal reference range but up to the upper limit may also be related to an increased preeclampsia–eclampsia risk. Women with hypothyroxinemia had an increased risk of preeclampsia–eclampsia, particularly for women with persistent hypothyroxinemia from the first to second trimesters. In addition, there is a dose–response relationship between hypothyroxinemia and preeclampsia–eclampsia risk; i.e., preeclampsia–eclampsia risk increased as the severity of hypothyroxinemia increased. In contrast, there was no association between FT4 levels or hypothyroxinemia and gestational hypertension risk.

Previous epidemiological studies regarding hypothyroxinemia generally focused on its influence on offspring neurodevelopment (5, 30–32). To date, however, studies investigating the association of hypothyroxinemia with perinatal outcomes are still limited, and the results are conflicting. Casey and colleagues reported no association of hypothyroxinemia with the risk of any perinatal outcomes (15). Two recent systematic reviews (33) showed that hypothyroxinemia in women was significantly associated with a higher risk of preterm birth (34) and a lower risk of SGA (35), respectively. Similar to HDPs, both of these outcomes are placenta-derived complications of pregnancy. A systematic review (2021) included nine studies that investigated the association of hypothyroxinemia and HDPs and reported no association of hypothyroxinemia with preeclampsia, but reported an association with gestational hypertension (13). However, only two of nine studies investigated the association of preeclampsia–eclampsia and hypothyroxinemia, with one study including eclampsia but not preeclampsia (12). A recent cohort study including a total of 1,108 women with hypothyroxinemia indicated that hypothyroxinemia (FT4 levels lower than the 5th percentile) is associated with an increased risk of preeclampsia (14). However, the study did not estimate the association of hypothyroxinemia with gestational hypertension risk.

Interestingly, we observed a different relationship of hypothyroxinemia with the risk of preeclampsia–eclampsia and gestational hypertension. Gong and colleagues reported that women with isolated hypothyroxinemia only during the second trimesters, but not in the first trimesters, had an increased risk of gestational hypertension (12). Another study performed in China reported that there is an inverse association between FT4 concentration in the third trimester but not in the first trimester and the risk of preeclampsia (36). The study also indicated that women diagnosed with hypothyroxinemia in the third trimester of pregnancy had an increased risk of developing preeclampsia, but not gestational hypertension, which is in line with our study. In normal pregnancy, hCG acts as a TSH-like agonist on the thyroid gland, and would lead to lower TSH and higher FT4 levels. We speculated that hypothyroxinemia diagnosed only in the first trimester but not in the second and third trimesters represented an attenuated hCG-induced increase in FT4, but could not lead to impairment of pregnancy. An experimental study by Wistar rats also indicated that the decidua responds to thyroid hormones in a gestational age-dependent manner (37). It remains on debate whether gestational hypertension and preeclampsia–eclampsia are distinct entities with shared manifestations, or different spectrum of the same disease. Previous studies suggested distinct cytokine profiles and different pathogenesis for gestational hypertension and preeclampsia (38, 39). Another study also showed that gestational hypertension and preeclampsia shared most risk factors, such as obesity and nullipara; however, the effect size showed a substantial difference (40). While future studies are warranted to determine the mechanisms underlying the different relationship of hypothyroxinemia with preeclampsia–eclampsia and gestational hypertension.

Vascular dysfunction and abnormal hemodynamics are common features of HDP (41). A population-based prospective study suggested that FT4 levels in early pregnancy were positively linearly associated with the umbilical artery pulsatility index (PI) in the second and third trimesters as well as with the uterine artery resistance index (RI) in the second trimester and the risk of uterine artery notching in the third trimester (42). Kilby and colleagues also observed that the serum concentration of FT4 was lower in fetus affected by intrauterine growth restriction (IUGR), but serum TSH levels were not significantly different (43). Both IUGR and preeclampsia are typically characterized by defective placentation eliciting inadequate uteroplacental blood perfusion and ischemia (44–46). An *in vitro* study elucidated that epidermal growth factor and 3,5,3’-triiodothyronine (T3) may act synergistically to regulate both the proliferation and differentiation of human trophoblasts (47). However, further studies are still required to identify the mechanism of different effects of hypothyroxinemia on the risk of preeclampsia–eclampsia and gestational hypertension.

To the best of our knowledge, this was the first study to elucidate a U-shaped association between FT4 levels and the risk of preeclampsia–eclampsia. The strengths of our study include the large sample size and extensive statistical analyses. Our study also had several limitations. First, during the study period, the diagnosis of preeclampsia–eclampsia might change; however, the diagnosis of preeclampsia–eclampsia is not related to the hypothyroxinemia and misclassification should be nondifferential, and the estimates were biased toward the null. Second, as an observational study, our findings demonstrated an association, not causality. Thyroid function assessment in our study mostly occurred in the first half of pregnancy, preceding the diagnosis of preeclampsia–eclampsia. Combined with our results on the dose–response relationship of FT4 levels and the risk of preeclampsia–eclampsia, our study met three main constitutions of causal reference according to Hill’s viewpoint, including temporality, dose–response, and consistency (48). Future studies with carefully controlled setting will need to determine the causal relationship and efficiency of levothyroxine treatment for hypothyroxinemia to reduce risk of preeclampsia–eclampsia. Third, as an observational study, we acknowledge that residual and unmeasured confounding is still possible in our study. Last, we only included women with live-born infants in the study, which might introduce selection bias and bias the estimation towards the null again.

## Conclusion

In conclusion, our findings suggested that hypothyroxinemia was associated with an increased risk of preeclampsia–eclampsia but not with gestational hypertension. Obstetrics should be aware of the potential increase in the risk of preeclampsia–eclampsia due to lower free thyroxine levels.

## Data Availability Statement

The raw data supporting the conclusions of this article will be made available by the authors, without undue reservation.

## Ethics Statement

The studies involving human participants were reviewed and approved by the Human Ethics Committee of Shanghai First Maternity and Infant Hospital. Written informed consent for participation was not required for this study in accordance with the national legislation and the institutional requirements.

## Author Contributions

QD, XS, and TD contributed to the conception and planning. GL, SH, YL, and XL contributed to carrying out the study. XS was involved in data analysis and drafted the article. All authors were involved in interpreting the data and critically reviewing the article. All authors gave approval of the final version for publication.

## Funding

This work was supported by the Shanghai Pu-dong Municipal Health Commission (grant number PW2019D-9) and the Shanghai Hospital Development Centre (grant number SHDC2020CR6021).

## Conflict of Interest

The authors declare that the research was conducted in the absence of any commercial or financial relationships that could be construed as a potential conflict of interest.

## Publisher’s Note

All claims expressed in this article are solely those of the authors and do not necessarily represent those of their affiliated organizations, or those of the publisher, the editors and the reviewers. Any product that may be evaluated in this article, or claim that may be made by its manufacturer, is not guaranteed or endorsed by the publisher.
