# Supplementary material for: Associations of Hypothyroxinemia With Risk of Preeclampsia–Eclampsia and Gestational Hypertension
Source: Front Endocrinol (Lausanne). 2021 Nov 4;12:777152. doi: 10.3389/fendo.2021.777152 (PMC8600315; doi:10.3389/fendo.2021.777152)
Supplement: Supplementary file 1 [file Table_1.docx]

**Supplementary Tables**

Supplementary Table S1. Normal reference ranges for TSH and FT4 levels

Supplementary Table S2. Association between hypothyroxinaemia and preeclampsia-eclampsia risk by the time of hypothyroxinaemia diagnosis

Supplementary Table S3. Association between hypothyroxinaemia and preeclampsia-eclampsia risk among women with optimal pre-pregnancy BMI

Supplementary Table S4. Association between hypothyroxinaemia and preeclampsia-eclampsia risk among women with negativity TPOAb

Supplementary Table S5. Association between hypothyroxinaemia and preeclampsia-eclampsia risk among women with TSH levels lower than 2.5mIU/L

Supplementary Table S6. Association between hypothyroxinaemia and preeclampsia-eclampsia risk by propensity score matching

Supplementary Table S1. Normal reference ranges for TSH and FT4 levels

|  | First trimester | Second trimester | Third trimester |
| --- | --- | --- | --- |
| FT4(pmol/L) |  |  |  |
| The 2.5^th^ percentile | 13.12 | 11.98 | 10.53 |
| The 5^th^ percentile | 13.61 | 12.45 | 11.05 |
| The 10^th^ percentile | 14.25 | 13.03 | 11.64 |
| The 97.5^th^ percentile | 22.53 | 20.7 | 18.58 |
| TSH (mIU/L) |  |  |  |
| The 2.5^th^ percentile | 0.05 | 0.02 | 0.16 |
| The 97.5^th^ percentile | 3.5 | 3.73 | 4.42 |

Supplementary Table S2. Association between hypothyroxinaemia and preeclampsia-eclampsia risk by the time of hypothyroxinaemia diagnosis

|  | N (%) | Crude OR  (95%CI) | Adjusted OR  (95%CI) ^*^ | *P* Value |
| --- | --- | --- | --- | --- |
| Euthyroid | 1967 (3.56) | 1.00 | 1.00 |  |
| ***Only during the first trimester*** | | | | |
| Hypothyroxinemia | 148 (3.62) | 1.04 (0.88-1.23) | 1.08 (0.91-1.27) | 0.389 |
| Mild | 64 (3.04) | 0.87 (0.68-1.11) | 0.90 (0.70-1.15) | 0.382 |
| Moderate | 45 (4.46) | 1.30 (0.97-1.74) | 1.33 (0.99-1.80) | 0.063 |
| Severe | 39 (3.98) | 1.16 (0.85-1.59) | 1.21 (0.88-1.66) | 0.240 |
| Trend by severity | / | 1.05 (0.97-1.14) | 1.07 (0.98-1.16) | 0.120 |
| ***Only during the second trimester*** | | | | |
| Hypothyroxinemia | 165 (3.90) | 1.09 (0.93-1.27) | 1.12 (0.96-1.30) | 0.165 |
| Mild | 73 (3.45) | 1.00 (0.80-1.25) | 1.01 (0.81-1.26) | 0.961 |
| Moderate | 42 (3.96) | 1.08 (0.80-1.46) | 1.12 (0.83-1.51) | 0.454 |
| Severe | 50 (4.74) | 1.25 (0.96-1.64) | 1.32 (1.01-1.73) | 0.046 |
| Trend by severity | / | 1.06 (0.98-1.14) | 1.08 (1.00-1.16) | 0.053 |
| ***Both the first and the second trimesters*** | | | | |
| Hypothyroxinemia | 18 (4.04) | 1.32 (0.99-1.76) | 1.37 (1.03-1.83) | 0.031 |
| Mild | 4 (2.35) | 0.79 (0.44-1.45) | 0.84 (0.46-1.53) | 0.561 |
| Moderate | 6 (5.61) | 1.60 (0.95-2.70) | 1.64 (0.97-2.78) | 0.065 |
| Severe | 8 (4.85) | 1.65 (1.09-2.49) | 1.70 (1.12-2.58) | 0.013 |
| Trend by severity | / | 1.18 (1.04-1.33) | 1.19 (1.05-1.35) | 0.005 |

*. Adjusted for parity, age, foetus sex, original of residence, serum ferritin, thyrotropin level, and TPOAb status.

Supplementary Table S3. Association between hypothyroxinaemia and preeclampsia-eclampsia risk among women with optimal prepregnancy BMI

|  | N (%) | Crude OR  (95%CI) | Adjusted OR  (95%CI) ^*^ | *P* Value |
| --- | --- | --- | --- | --- |
| Euthyroid | 1170 (3.25) | 1.00 | 1.00 |  |
| Hypothyroxinemia | 144 (3.23) | 1.15 (1.00-1.32) | 1.22 (1.06-1.40) | 0.005 |
| Mild | 67 (2.94) | 1.00 (0.81-1.21) | 1.05 (0.86-1.29) | 0.644 |
| Moderate | 36 (3.33) | 1.18 (0.91-1.54) | 1.26 (0.97-1.64) | 0.085 |
| Severe | 41 (3.74) | 1.41 (1.11-1.77) | 1.50 (1.19-1.90) | 0.001 |
| Trend by severity |  | 1.10 (1.03-1.18) | 1.13 (1.06-1.21) | ＜0.001 |

*. Adjusted for parity, age, foetus sex, original of residence, serum ferritin, thyrotropin level, and TPOAb status.

Supplementary Table S4. Association between hypothyroxinaemia and preeclampsia-eclampsia risk among women with negativity TPOAb

|  | N (%) | Crude OR  (95%CI) | Adjusted OR  (95%CI) ^*^ | *P* Value |
| --- | --- | --- | --- | --- |
| Euthyroid | 1656 (3.56) | 1.00 | 1.00 |  |
| Hypothyroxinemia | 211 (3.72) | 1.06 (0.92-1.21) | 1.09 (0.95-1.25) | 0.213 |
| Mild | 85 (2.96) | 0.84 (0.68-1.03) | 0.86 (0.70-1.94) | 0.155 |
| Moderate | 62 (4.39) | 1.27 (1.00-1.61) | 1.29 (1.01-1.64) | 0.043 |
| Severe | 64 (4.60) | 1.30 (1.03-1.64) | 1.36 (1.07-1.72) | 0.011 |
| Trend by severity |  | 1.07 (1.01-1.15) | 1.09 (1.02-1.17) | 0.010 |

*. Adjusted for parity, age, prepregnancy BMI, foetus sex, original of residence, serum ferritin, thyrotropin levels.

Supplementary table S5. Association between hypothyroxinaemia and PEE risk among women with TSH lower than 2.5mIU/L

|  | N (%) | Crude OR  (95%CI) | Adjusted OR (95%CI) ^*^ | *P* Value |
| --- | --- | --- | --- | --- |
| Euthyroid | 1750 (3.57) | 1.00 | 1.00 |  |
| Hypothyroxinemia | 225 (3.84) | 1.16 (1.03-1.31) | 1.20 (1.07-1.35) | 0.003 |
| Mild | 97 (3.29) | 1.00 (0.84-1.18) | 1.03 (0.86-1.22) | 0.785 |
| Moderate | 63 (4.35) | 1.33 (1.07-1.65) | 1.36 (1.10-1.69) | 0.005 |
| Severe | 65 (4.44) | 1.32 (1.07-1.63) | 1.37 (1.12-1.69) | 0.003 |
| Trend by severity |  | 1.10 (1.04-1.17) | 1.12 (1.06-1.19) | <0.001 |

*. Adjusted for parity, age, prepregnancy BMI, fetus sex, original of residence, serum ferritin, and TPOAb status.

Supplementary Table S6. Association between hypothyroxinaemia and preeclampsia-eclampsia (PEE) risk by propensity score matching

|  | Overall PEE |  | Early-onset PEE |  | Late-onset PEE |
| --- | --- | --- | --- | --- | --- |
|  | OR（95%CI） |  | OR（95%CI） |  | OR（95%CI） |
| Euthyroid | 1.00 |  | 1.00 |  | 1.00 |
| Hypothyroxinemia | 1.02 (0.83-1.24) |  | 4.50 (0.97-20.83) |  | 0.98 (0.80-1.20) |
| Mild | 0.81 (0.61-1.09) |  | 4.00 (0.45-35.78) |  | 0.78 (0.58-1.05) |
| Moderate | 1.26 (0.84-1.89) |  | 2.05 (0.18-21.80) |  | 1.27 (0.84-1.91) |
| Severe | 1.21 (0.84-1.75) |  | 8.12 (1.49-44.39) |  | 1.13 (0.78-1.64) |
| Trend by severity | 1.08 (0.98-1.18) |  | 1.79 (1.09-2.95) |  | 1.06 (0.96-1.16) |
